# Nonlinear associations of BMI and body dissatisfaction with depression and an exploratory decomposition of the indirect association: a cross-sectional study of Chinese university students

**DOI:** 10.3389/fpsyt.2026.1916755

**Published:** 2026-09-09

**Authors:** Lanqier Li, Hongfei Yu, Guifang Su, QiuJun Wang, Feng Chen, Linlin Yue

**Affiliations:** 1 School of Public Health and Health Management, Gannan Medical University, University Park, Rongjiang New Area, Ganzhou, Jiangxi, China; 2 The First Affiliated Hospital of Gannan Medical University, Zhanggong District, Ganzhou, Jiangxi, China

**Keywords:** body dissatisfaction, body mass index, depression, exploratory mediation analysis, indirect association, non-linear association

## Abstract

**Objective:**

This study aimed to examine the non-linear associations of body mass index (BMI) and body dissatisfaction with depressive symptoms among Chinese university students, and to explore how the indirect association between BMI and depressive symptoms through body dissatisfaction varies across the BMI range.

**Methods:**

A cross-sectional design was used, and 7,928 full-time students were recruited from eight universities in China. Depressive symptoms were assessed with the Zung Self-Rating Depression Scale (SDS); body dissatisfaction was operationalised as the absolute difference between measured BMI and self-reported ideal BMI. Analyses comprised restricted cubic spline (RCS) models, an exploratory non-linear decomposition of the indirect association, generalised additive models (GAM) as a sensitivity analysis, a supplementary receiver operating characteristic (ROC) analysis, and k-means clustering. Because measured BMI enters the calculation of body dissatisfaction, the exposure and the proposed mediator are not mathematically independent; all decomposition results are therefore reported as exploratory.

**Results:**

Both BMI and body dissatisfaction were non-linearly associated with depressive symptoms (both P < 0.01). In the exploratory decomposition, the estimated indirect association between BMI and depressive symptoms through body dissatisfaction attenuated progressively as BMI increased. In the supplementary ROC analysis, the BMI value maximising the Youden index was 23.11 kg/m² (AUC = 0.768); this is a sample-specific, internally derived discrimination point rather than a screening threshold. A five-stratum descriptive matrix combining BMI groups with body-dissatisfaction clusters was constructed, and the observed prevalence of depressive symptoms ranged from 14.4% to 100.0% across strata.

**Conclusion:**

BMI and body dissatisfaction were non-linearly associated with depressive symptoms in this sample, and the magnitude of the indirect association through body dissatisfaction varied across the BMI range. Because the body-dissatisfaction indicator is mathematically derived from measured BMI and the design was cross-sectional, these results describe statistical associations and should not be read as evidence of a causal mediating mechanism. The risk-stratification matrix was developed and evaluated in the same dataset without internal or external validation; it is therefore exploratory and requires independent validation before any screening application.

## 1 Introduction

As an increasingly prominent global public health challenge, depression not only severely undermines individuals’ physical and mental well-being but also poses a significant threat to the growth and development of young people.

Depression is defined as a mood disorder with core symptoms including persistent low mood, loss of interest, changes in sleep or appetite, fatigue, diminished self-worth, difficulty concentrating, and suicidal ideation (1). According to data from the World Health Organization, approximately 332 million people worldwide suffer from depression (2); other studies have indicated that the incidence of depression is highest among young people aged 18 to 25 years (approximately 17%), and the university student population falls within this high-risk age group (3, 4). A study conducted in China involving 2,580 university students confirmed that 43.6% of the participants exhibited symptoms of depression, with symptoms being more pronounced among students from urban areas (5). This series of data clearly reveals that depression has become highly prevalent among college students, necessitating focused attention in the field of public health and providing a concrete basis for formulating targeted intervention strategies.

Meanwhile, the global age-standardized obesity rate has shown a sharp upward trend from 1975 to 2016 (6), and a study in the United States found that among the college student population, the obesity rate steadily increased from freshmen to seniors (rising from 36% to 55% for males and from 32% to 46% for females) (7). A study in China likewise confirmed that the average body mass index (BMI) of Chinese college students has increased overall, rising from approximately 21.85 in 2014 to over 22.70 in 2023, with the rate of BMI growth accelerating after 2020 (8). The aforementioned data collectively indicate that obesity and depression have become pressing global public health crises, urgently requiring a coordinated response through multifaceted measures, such as health education, policy interventions, and guidance on individual behaviour.

In the context of global public health in the 21st century, the intricate relationship between BMI and depression highlights the formidable challenges faced by human physical and mental health. Although numerous studies have consistently demonstrated a significant association between BMI and depression, for example, a study involving 10,686 adults in Shenzhen, China, found a pronounced ‘U-shaped’ relationship between BMI and depression (9), and similar findings have been validated in studies conducted in the Netherlands (10).

However, we were unable to determine precisely which intrinsic psychological dynamics underlie the increased risk of depression. This cognitive gap limits the translation of the research findings into effective intervention strategies. If we cannot gain insight into the individual psychological realities associated with BMI, risk assessments based on BMI will appear crude and lack specificity, as psychological dimensions are not taken into account.

Against this background, we shifted the focus of our study from the overall association between BMI and depression to a candidate psychological correlate linking the two, namely body dissatisfaction. This refers to a negative subjective experience of discontent with one’s body shape, form, or weight (11). Body dissatisfaction typically arises from the gap between an individual’s perceived actual physical state and the socially idealized body shape. Previous research has reported an association between body dissatisfaction and depression. For example, a study of young adults in Pakistan found a positive correlation between body dissatisfaction and depression (12). Research among university students in southern China likewise reported that greater dissatisfaction with body image was accompanied by higher levels of depressive symptoms, with a stronger association among female than male students (13).

However, previous studies have provided limited insight into whether body dissatisfaction links BMI to depression in a non-linear manner. Social comparison theory suggests that individuals evaluate their bodies against culturally valued standards, such that deviations from an internalized ideal may increase unfavourable appearance comparisons and body dissatisfaction (14, 15). Self-discrepancy theory further proposes that a perceived gap between the actual and the ideal body may elicit negative affect (16). Importantly, the psychological impact of this discrepancy may vary across its severity, because individuals may adopt different body-image coping responses, including avoidance and positive rational acceptance (17). Increases in body dissatisfaction may therefore not correspond to proportional increases in depressive symptoms.

In summary, the present study set out to move beyond the linear assumption and to characterize the non-linear associations of BMI and body dissatisfaction with depressive symptoms; to examine, on an exploratory basis, how the indirect association through body dissatisfaction differs across the BMI range; and to describe, again on an exploratory basis, how depressive-symptom prevalence is distributed when BMI groups and body-dissatisfaction clusters are cross-classified. The intended contribution is descriptive and hypothesis-generating: the analyses are intended to inform the design of future longitudinal studies rather than to provide a validated screening instrument.

Accordingly, the present study examined the nonlinear associations among BMI, body dissatisfaction, and depressive symptoms in Chinese university students. Based on the preceding theoretical and empirical evidence, we proposed the following hypotheses: H1, BMI would be nonlinearly associated with depression; H2, body dissatisfaction would be nonlinearly associated with depression; and H3, the indirect association between BMI and depression through body dissatisfaction would vary across BMI levels. Given the cross-sectional design and the inclusion of measured BMI in the calculation of body dissatisfaction, H3 was examined as an exploratory hypothesis rather than as evidence of a causal mediating mechanism.

## 2 Methods

### 2.1 Sample size

The target sample size was prospectively estimated using G*Power version 3.1 before participant recruitment (18). For the restricted cubic spline regression model examining the relationship between BMI and depression levels, the significance level (α) was set at 0.05, the statistical power (1−*β*) at 0.80, and the effect size (*f²*) was specified as 0.02 to detect a small effect (19), with two predictor variables included. The results indicated that a minimum of 485 participants would be required to satisfy the statistical assumptions. A total of 7,928 participants were enrolled in this study, and the sample size was deemed sufficient to robustly identify nonlinear associations between BMI and depression levels. For the five-level risk stratification formed by the cross-classification of BMI-based groups and body dissatisfaction clusters in **Table 1**, it is an exploratory *post hoc* subgroup description, and no sample size sensitivity analysis was prespecified for each subgroup. This study quantifies the precision of its estimates by reporting the Wilson 95% confidence intervals of the prevalence in each subgroup.

**Table 1:** Observed prevalence of clinically significant depressive symptoms across combined BMI and body-dissatisfaction strata.

Risk level	Composite features	Total	Number of people with depression	Average level of depression	Probability of depression	CI_lower	CI_upper
Tier 1	Low BMI+Medium BD	4116	593	54.7	14.4%	13.4	15.5
Tier 2	High BMI+High BD	563	331	53.3	58.8%	54.6	62.9
Tier 3	Low BMI+High BD	1030	631	60.4	61.3%	58.2	64.2
Tier 4	High BMI+Low BD	453	288	45.0	63.6%	58.9	68
Tier 5	High BMI+Medium BD	1766	1766	60.4	100.0%	99.7	100

BD, Body Dissatisfaction.

### 2.2 Research design and participants

This study adopted a cross-sectional research design and selected eight comprehensive universities across five provinces and municipalities (Sichuan, Jiangxi, Hubei, Shanghai, and Anhui) to conduct the investigation, thereby comprehensively covering a diverse range of disciplines, including humanities, sciences, engineering, and medicine, to enhance the generalizability of the study. A multistage stratified cluster sampling method was employed during the sampling process to ensure the representativeness of the sample; however, this study was not preregistered.

Participant selection strictly adhered to standardized criteria; inclusion criteria were limited to full-time university students enrolled at various higher education institutions, who also had to voluntarily participate and complete the informed consent process in its entirety. Exclusion criteria included the presence of severe physical illnesses, a definite history of psychiatric disorders, or recent (within the past three months) use of psychotropic medications.

The recruitment and data collection for this study were conducted in an orderly and phased manner from September 2023 to December 2024. During the recruitment phase, the target population was invited to participate through official campus announcements; class group notifications; and coordinated collaborations with relevant school departments, with clear communication regarding the study’s objectives, core procedures, participant rights, and privacy protection measures, ensuring that participants voluntarily enrolled after being fully informed. In the survey phase, researchers organized participants to complete measurements and questionnaires at designated locations in batches to ensure orderly data collection. Standardized instruments, including the Seca 213 stadiometer and Tanita BC-610 body composition analyser, were used in the physical assessment segment to accurately measure core indicators, such as height, weight, and body composition. The questionnaire employed a dual-format approach using both paper and electronic versions, with trained researchers guiding the participants in person. Questionnaire completeness and logical consistency were immediately checked, and incomplete or contradictory responses were excluded to ensure the reliability of the collected questionnaire data.

A total of 8,500 questionnaires were submitted. During participant screening and data quality control, 572 questionnaires were excluded: 85 because the participants did not meet the eligibility criteria, 156 because of incomplete questionnaires or missing key variables, 78 because of duplicate submissions, 124 because of logically inconsistent responses, 59 because of implausible or unverifiable anthropometric measurements, and 70 because of other prespecified quality-control reasons. After these exclusions, 7,928 participants were included in the final analytical sample. The valid-questionnaire rate was 93.3%.

This study adhered to the ethical guidelines of the Declaration of Helsinki and was approved by the Ethics Committee of Gannan Medical University, China (Approval No. 2021110). All participants signed informed consent forms before the survey. The consent process emphasized the confidentiality of the data and their use for academic purposes, explicitly informing the participants of their right to withdraw at any time. To protect the participants’ privacy, all data were anonymized, and personal information was stored separately from the research data.

### 2.3 Measurement instruments and data collection

#### 2.3.1 Physical measurements

Height was measured using a portable stadiometer (Seca 213, Germany) accurate calibrated body composition analyser (Tanita BC-610, Japan) accurate to 0.1 kg. All measurements were uniformly scheduled between 19:00 and 21:00 from Monday to Sunday, with participants instructed to avoid vigorous exercise and heavy meals for two hours prior to measurement. All measurements were conducted with the participants wearing light indoor clothing and without shoes or socks.

#### 2.3.2 Depression

The Zung Self-Rating Depression Scale (SDS) was used to assess participants’ depression levels (20). The scale is based on clinical manifestations of depressive symptoms and consists of 20 self-rated items. Scoring was conducted using a 1–4 Likert scale, with higher scores indicating more severe depressive symptoms. The grouping criteria followed the established cutoff value of the scale (53 points): participants with a total score≥53 were classified into the depression group, while those with a total score <53 were classified into the non-depression group. As a classical tool for depression assessment, the SDS demonstrates strong suitability for quantitatively capturing subjective perceptions of depression. Previous research targeting Chinese university students has shown that this scale has good internal consistency reliability, with a Cronbach’s α coefficient of 0.85 (21), indicating that the use of this scale in the present study has a reliable psychometric basis.

In this study, the continuous SDS standard score was used as the primary outcome to assess depressive symptom severity and its potential nonlinear associations with BMI and body dissatisfaction. A higher SDS score indicated more severe depressive symptoms. Binary depressive symptoms, defined as an SDS standard score≥53, were used as a secondary outcome only for estimating the prevalence of depressive symptoms and constructing the combined risk stratification.

#### 2.3.3 BMI and body dissatisfaction

Using calibrated body composition analysers and electronic stadiometers, participants’ weight and height were measured to the nearest 0.1 kg and 0.1 cm, respectively. Measured BMI was calculated as weight in kilograms divided by height in meters squared. Participants’ self-reported ideal BMI was collected using the questionnaire. Body image dissatisfaction was operationalized using the BMI discrepancy method as the absolute difference between measured BMI and ideal BMI: body image dissatisfaction = |measured BMI-ideal BMI| (22). A higher discrepancy value represents a greater mismatch between an individual’ s measured and ideal body size. Because measured BMI is a mathematical component of this discrepancy score, this indicator may share structural variance with BMI and should not be regarded as fully independent of BMI. Accordingly, analyses involving this indicator, particularly the mediation analysis, were interpreted as exploratory.

### 2.4 Data quality control and outlier treatment

To ensure data quality and to limit the influence of a small number of extreme observations on the spline and polynomial models, outliers in BMI and body-dissatisfaction scores were identified and treated. Outlier identification used the boxplot rule (1.5 times the interquartile range) and the Z-score rule (|Z| > 3) in combination, and only observations flagged by both rules were treated. Extreme values were handled by 95% winsorisation: observations below the 2.5th percentile and above the 97.5th percentile were replaced by the corresponding percentile values. For BMI, 198 observations fell below the 2.5th percentile and 198 above the 97.5th percentile (5.0% of the sample in total). Winsorisation rather than deletion was chosen because the flagged values were biologically possible and because deletion would have discarded participants at the two ends of the BMI distribution, which are precisely the regions in which a non-linear association is of interest; retaining these observations untreated, by contrast, would have allowed a small number of points to exert disproportionate leverage on the tails of the spline fit, where data density is lowest. Because winsorisation compresses the observed range, the estimated associations at the extremes of the BMI distribution should be interpreted as conservative, and the treatment is reported here so that readers can judge its influence. All analyses reported below were performed on the winsorised data; no analysis was performed on the untreated data.

### 2.5 Data analysis

This study used the SDS standard score (continuous variable) as the primary outcome measure and an SDS score of ≥53 (binary variable) as the secondary outcome measure.

Statistical analyses were performed using SPSS 27.0 in conjunction with RStudio (4.2.1). Continuous variables were described as mean ± standard deviation, and categorical variables were described as frequency (percentage). Independent-samples t-tests were used for comparisons between groups, and multicollinearity was assessed using the variance inflation factor (VIF).

To identify non-linear relationships, restricted cubic splines (RCS) were used to model the associations of BMI and body dissatisfaction with depressive symptoms. The number and placement of knots were specified *a priori* following Harrell’s percentile-based recommendation and were not selected on the basis of model fit or of any exploratory inspection of the data: four knots were placed at the 5th, 35th, 65th and 95th percentiles of each variable’s distribution (for BMI, 17.05, 20.17, 23.12 and 37.16 kg/m²; for body dissatisfaction, 0.08, 2.63, 5.00 and 17.00). No alternative knot specification was fitted, and no knot location was moved after the models were estimated. The fit of the RCS model was compared with that of the corresponding linear model using a likelihood ratio test, with a significance level of α = 0.05.

Association and decomposition analyses were conducted using the SPSS Medcurve plugin. First, a multiple linear regression model was fitted to estimate the association between BMI and depressive-symptom severity. Because the RCS results indicated a non-linear trend, a non-linear decomposition of the indirect association was then performed, using the bootstrap method with 5,000 resamples to obtain 95% confidence intervals. The body-dissatisfaction score is the absolute difference between measured BMI and self-reported ideal BMI and is therefore mathematically derived, in part, from the exposure; the exposure and the proposed mediator are neither statistically nor conceptually independent. This analysis is consequently reported as an exploratory statistical decomposition of an indirect association and not as a test of a mediating mechanism. Temporal ordering between BMI, body dissatisfaction and depressive symptoms cannot be established in a cross-sectional design, and no causal interpretation is intended.

In the nonlinear mediation model, the instantaneous mediation effect is defined as the partial indirect association between BMI and depression via body dissatisfaction at a specific BMI value; it is calculated as the product of the instantaneous slope of the BMI→body dissatisfaction path and the coefficient of the body dissatisfaction→depression path. Since the BMI→body dissatisfaction path includes a quadratic term, its slope and mediation effect vary with changes in BMI.

This study selected three representative BMI values (one standard deviation below the mean, the mean, and one standard deviation above the mean) to estimate the instantaneous indirect effects. Statistical uncertainty was assessed using 5,000 Bootstrap resamples; results were considered statistically significant if the 95% confidence interval did not include 0.

To further validate the robustness of the nonlinear mediation model and explain the differences between the RCS and quadratic term model results, this study conducted a sensitivity analysis using a generalized additive model (GAM). Both BMI and body dissatisfaction were included in the model using penalized spline smoothing functions to estimate the trends in the instantaneous indirect effects at different BMI levels. The GAM analysis was performed using the mgcv package in RStudio.

With depression as a binary outcome variable, the predictive performance of BMI was evaluated using ROC curves; the AUC was calculated, the optimal cutoff value for BMI was determined based on the maximum Youden’ s index, and sensitivity, specificity, positive predictive value, and negative predictive value were reported.

Body dissatisfaction was used as the clustering variable for K-means clustering analysis. Since univariate clustering was employed, all observations were subjected to Euclidean distance calculation under the same dimension, and therefore the data were not standardized (Z-score) prior to clustering. The optimal number of clusters was determined using the elbow method by calculating the within-cluster sum of squares (WCSS) for k = 1 to 6 and identifying the k value corresponding to the inflection point. The K-means algorithm was implemented using the kmeans() function in RStudio: the initial cluster centers were randomly initialized; to reduce the risk of local optima, 25 random starts (nstart = 25) were set, and the result with the minimum WCSS was selected; the maximum number of iterations was set to 10, and all actual computations converged within this range. To ensure reproducibility, the random seed was set using set.seed(123). Based on the mean levels of body dissatisfaction in each cluster, participants were divided into three groups: low, medium, and high. A χ² test was used to analyze the association between BMI risk groups and clustering results.

Finally, BMI groups defined by the supplementary ROC-derived cut-off and the body-dissatisfaction clusters were cross-classified, and the observed prevalence of depressive symptoms was calculated within each resulting stratum, together with Wilson 95% confidence intervals. This matrix is a descriptive summary of the observed data. It was derived and evaluated in the same dataset; no internal validation, cross-validation, bootstrap optimism correction or external validation was undertaken, and no independent cohort was available. Any apparent discriminative performance is therefore optimistic by construction, and the matrix is presented as an exploratory description rather than as a prediction model.

Missing data were handled by complete-case analysis. Questionnaires with missing values on key variables were excluded at the data-screening stage as described in Section 2.2, and no imputation method was applied to the analytical sample of 7,928 participants; all participants included in the analyses had complete data on BMI, ideal BMI, SDS score, age and sex.

Throughout the Results, effect estimates are reported alongside P values. Given the large sample size, statistical significance was not treated as evidence of clinical importance, and interpretation is based on the magnitude of the estimates and the width of their confidence intervals rather than on P values alone.

## 3 Results

The participants’ characteristics are listed in **Table 2**. This study included 7,928 Chinese university students. Regarding the sex composition of the sample, there were 3,237 males (40.83%) and 4,691 females (59. 17%). The average age of participants was 18.98 ± 1.30 years (Mean ± SD), with the majority aged between 17 and 22 years. The mean BMI was 23.16 ± 6.55 kg/m², with 28.51% of individuals classified as overweight or obese (BMI≥24 kg/m²) and 16.45% classified as underweight (BMI < 18.5 kg/m²). In terms of psychological and behavioural indicators, the mean SDS standard score was 50.77 ± 10.89, and the mean body dissatisfaction score was 5.32 ± 5.29.

**Table 2:** Participant characteristics (n=7,928).

Characteristics	Mean ± SD or n(%)			*P*
	Male(n=3,237)	Female(n=4,691)	Total(n=7,928)	
Age	19.24 ± 1.50	18.80 ± 1.11	18.98 ± 1.30	< 0.01
Depression Level	50.37 ± 11.50	51.05 ± 10.43	50.77 ± 10.89	< 0.01
BMI(kg/m²)	23.50 ± 6.78	22.93 ± 6.37	23.16 ± 6.55	< 0.05
Underweight	516(15.94)	788(16.80)	1304(16.45)	0.296
Normal	1744(53.88)	2619(55.83)	4363(55.04)	< 0.01
Overweight and Obese	977(30.18)	1284(27.37)	2261(28.51)	0.298
Body Dissatisfaction(kg/m²)	5.60 ± 5.73	5.12 ± 4.96	5.32 ± 5.29	< 0.01

Independent-samples t-tests were used to compare the continuous SDS standard score and body dissatisfaction score between male and female participants (**Table 2**). Female participants had a higher mean SDS standard score than male participants (P<0.01), whereas male participants had a higher mean body dissatisfaction score than female participants (P<0.01).

After applying a 95% winsorisation, the standard deviation of BMI decreased from 6.41 to 5.75 (with the mean adjusted from 23.15 to 23.01, a change of less than 1%), and the standard deviation of body dissatisfaction scores decreased from 5.29 to 4.84 (with the mean adjusted from 5.32 to 5.20, a change of less than 3%). The winsorisation effectively reduced data dispersion, while the core central tendency remained essentially unchanged.

### 3.1 Factors influencing depression level

The results of the hierarchical multiple linear regression analysis are shown in **Table 3**. In Model 1, which was unadjusted, both BMI and body dissatisfaction were positively associated with the SDS score (both P < 0.01). After adjustment for age and sex in Model 2, both associations persisted: BMI (B = 0.721, standardised β = 0.425, P < 0.01) and body dissatisfaction (B = 0.177, standardised β = 0.086, P < 0.01). Age was inversely associated with the SDS score (B = -0.556, standardised β = -0.067, P < 0.01), and female students scored higher than male students (B = 0.905, standardised β = 0.041, P < 0.01). The variance inflation factor was below 2 for all variables, indicating no material multicollinearity. The standardised coefficients indicate that, apart from BMI, the associations were small in magnitude: a one-standard-deviation increase in body dissatisfaction corresponded to an increase of less than one-tenth of a standard deviation in the SDS score, and the age and sex coefficients were smaller still. These estimates reach significance partly because of the sample size and should not be read as indicating clinically meaningful differences at the individual level. Models were adjusted for age and sex only; no other covariate was available.

**Table 3:** Factors influencing college students’ depression level.

Variables	Model 1				Model 2			
	B	β	*P*	*VIF*	B	β	*P*	*VIF*
Constant	33.281	–	< 0.01	–	42.251	–	< 0.01	
BMI(kg/m²)	0.715	0.421	< 0.01	1.609	0.721	0.425	< 0.01	1.610
Body Dissatisfaction	0.175	0.085	< 0.01	1.609	0.177	0.086	< 0.01	1.610
Age	–	–	–	–	-0.556	-0.067	< 0.01	1.029
Gender	–	–	–	–	0.905	0.041	< 0.01	1.030

VIF, Variance Inflation Factor.

### 3.2 Restricted cubic spline model analysis of BMI and body dissatisfaction on depression

**Figure 1:**
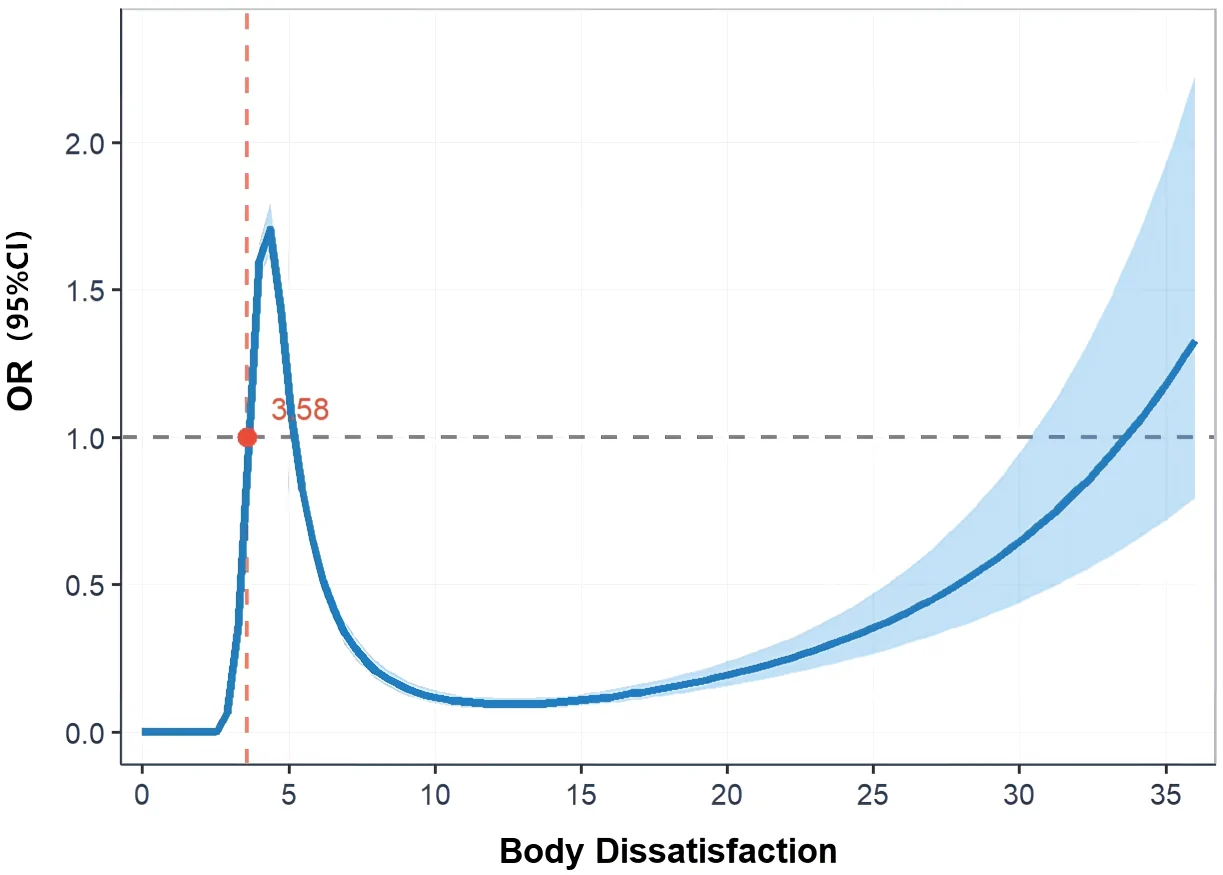
Nonlinear dose-response relationship between body dissatisfaction and depression.

**Figure 2:**
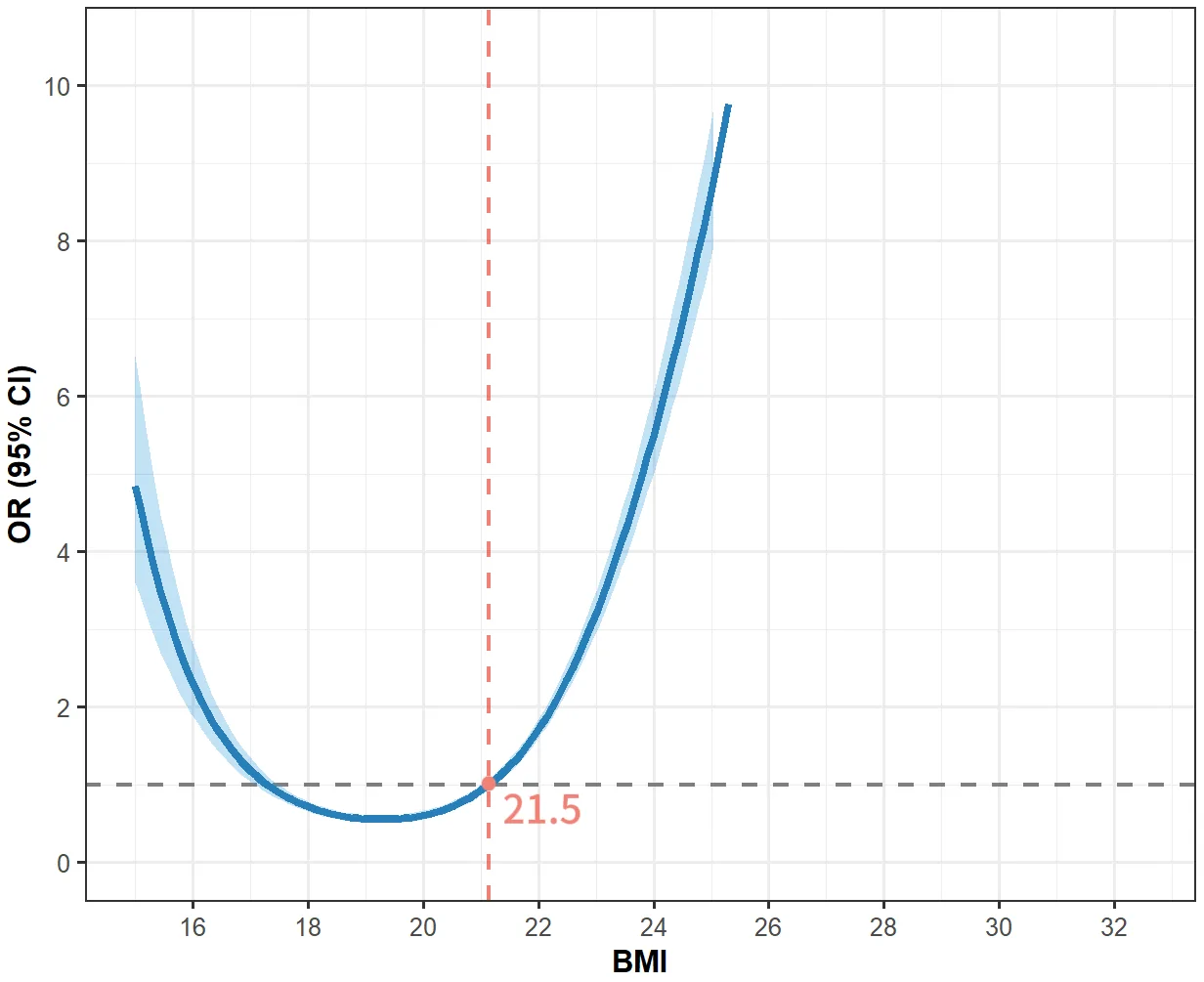
Nonlinear dose-response relationship between body mass index (BMI) and depression.

Through a logistic RCS analysis, we examined the associations of BMI and body dissatisfaction with levels of depression. The results showed a significant nonlinear association between body dissatisfaction and odds of clinically significant depressive symptoms (P<0.01, **Figure 1**; **Table 4**), and the relationship between BMI and odds of clinically significant depressive symptoms was not linear, but rather exhibited a nonlinear effect (P<0.01, **Figure 2**; **Table 5**). In the RCS analysis, a BMI of 21.5 kg/m^2^ was specified as the reference value, at which the odds ratio was normalized to 1. This reference value served only as the baseline for expressing relative odds along the spline curve and should not be interpreted as an optimal diagnostic or risk-classification cutoff.

**Table 4:** The nonlinear relationship between body dissatisfaction and depression.

Variables	*χ²*	df	*P*
Body Dissatisfaction	1005.07	3	< 0.01
Nonlinear	995.87	2	< 0.01
Total	1005.07	3	< 0.01

**Table 5:** The nonlinear relationship between body mass index (BMI) and depression.

Variables	*χ²*	df	*P*
BMI	1461.17	3	< 0.01
Nonlinear	450.75	2	< 0.01
Total	1461.17	3	< 0.01

### 3.3 Exploratory non-linear decomposition of the indirect association between BMI and depressive symptoms via body dissatisfaction

The RCS models described above indicated a non-linear association between BMI and depressive symptoms. Because the SPSS Medcurve plugin does not support spline functions, a quadratic polynomial specification was used in this section to describe the shape of the association and to decompose the indirect component. Results, adjusted for sex, are presented in **Tables 6**, **7**. These estimates are exploratory and are not interpreted as mediating effects.

**Table 6:** The pathway effect of BMI on body dissatisfaction.

Variables	Coeff	SE	t	*P*
Constant	-6.6009	0.5722	-11.5368	< 0.01
BMI	0.5431	0.0380	14.2809	< 0.01
BMI^2^	-0.0006	0.0006	-0.9675	0.3333
Gender	-0.1990	0.0955	-2.0832	0.0373

**Table 7:** The pathway effect of body dissatisfaction on depression.

Variables	Coeff	SE	t	*P*
Constant	20.7791	1.1677	17.7951	<0.01
BMI	0.8989	0.0839	10.7177	<0.01
BMI^2^	0.0021	0.0014	1.4938	0.1353
Body Dissatisfaction	2.5325	0.0501	50.5601	<0.01
Body Dissatisfaction^2^	-0.1203	0.0023	-51.9792	<0.01
Gender	0.8123	0.1795	4.5251	<0.01

BMI^2^ represents the squared term of Body Mass Index, and Body Dissatisfaction^2^ represents the squared term of body dissatisfaction scores, used to examine nonlinear associations.

As shown in **Table 6**, the linear term of BMI was positively associated with body dissatisfaction (B = 0.5431, SE = 0.0380, 95% CI 0.4686 to 0.6176, P < 0.01), whereas the quadratic term was not statistically significant (B = -0.0006, SE = 0.0006, 95% CI -0.0018 to 0.0006, P = 0.3333), indicating that a linear specification was adequate for the BMI-body dissatisfaction association in this model. Wald 95% confidence intervals are reported alongside the coefficients so that the precision of each estimate can be judged independently of its P value.

As shown in **Table 7**, both the linear and the quadratic terms of body dissatisfaction were statistically significant (linear: B = 2.5325, SE = 0.0501, 95% CI 2.4343 to 2.6307; quadratic: B = -0.1203, SE = 0.0023, 95% CI -0.1248 to -0.1158; both P < 0.01), whereas the quadratic term of BMI was not (B = 0.0021, SE = 0.0014, 95% CI -0.0006 to 0.0048, P = 0.1353), indicating that the BMI-depressive symptom association was adequately captured by a linear term within this quadratic specification (B = 0.8989, SE = 0.0839, 95% CI 0.7345 to 1.0633). This does not contradict the RCS finding of a non-linear association: the two models rest on different assumptions. A single quadratic term can represent only a symmetric U-shaped or inverted U-shaped relationship and has limited power to detect asymmetric curvature, whereas RCS permits a more flexible representation of complex and asymmetric associations. The divergence should therefore be read as a difference in model flexibility rather than as evidence for or against non-linearity.

The estimated instantaneous indirect associations are presented in **Table 8**. The indirect association between BMI and depressive symptoms through body dissatisfaction decreased monotonically as BMI increased: it was largest at the lower BMI level (BMI 16.74; θ= 1.0729, 95% CI 0.9171 to 1.2455), smaller at the mean BMI level (BMI 23.15; θ= 0.6438, 95% CI 0.5943 to 0.6934) and smallest at the higher BMI level (BMI 29.56; θ= 0.2327, 95% CI 0.1911 to 0.2749). The bootstrap 95% confidence intervals excluded zero at all three levels. Because measured BMI is a component of the body-dissatisfaction score, these quantities describe a statistical decomposition of shared variance and are not estimates of a mediated causal effect.

**Table 8:** Results of the instantaneous indirect effect test.

Pathway	Representative level	XVAL	θ	LowerCI	UpperCI
BMI→Body dissatisfaction→Depression	M-1SD	16.7370	1.0729	0.9171	1.2455
	Mean	23.1506	0.6438	0.5943	0.6934
	M+1SD	29.5641	0.2327	0.1911	0.2749

Taken together, the RCS and decomposition results indicate that the overall association between BMI and depressive symptoms departs from linearity, and that the estimated indirect component acting through body dissatisfaction is larger at lower BMI values than at higher ones. Because the mediator is mathematically derived from the exposure and the data are cross-sectional, it cannot be determined from these analyses whether the observed curvature originates in a psychological pathway, in the shared variance between BMI and the discrepancy score, or in both.

To assess the robustness of the quadratic specification and to clarify the source of the asymmetry in the RCS curve, a sensitivity analysis was performed using a generalised additive model (GAM) to estimate the instantaneous indirect association across BMI levels. The estimated indirect association declined continuously as BMI increased: below approximately 26.5 kg/m² it was positive (range 3.329 to 0.004), and above approximately 26.5 kg/m² it approached and then crossed zero (range -0.014 to -0.102). These GAM results are consistent with the asymmetric pattern seen in the RCS curve and indicate that the declining trend observed in the quadratic model within the BMI range below 26.5 kg/m²is not an artefact of the quadratic specification. The GAM was fitted in the same dataset and therefore provides internal consistency rather than independent validation.

### 3.4 Supplementary ROC curve analysis

In the supplementary ROC analysis, the AUC for BMI in discriminating between participants with and without clinically significant depressive symptoms was 0.768. The BMI cutoff that maximized the Youden index was 23.11 kg/m^2^. This value represents a sample-specific statistical discrimination point for the binary SDS outcome and should not be interpreted as a diagnostic threshold for depression or as the turning point of the nonlinear BMI–depression association. The ROC-derived cutoff of 23.11 kg/m^2^ served a different statistical purpose from the RCS reference value of 21.5 kg/m^2^, which was used only to normalize the odds ratio to 1; neither value constitutes a diagnostic threshold for depression.

### 3.5 Exploratory risk stratification

**Figure 3:**
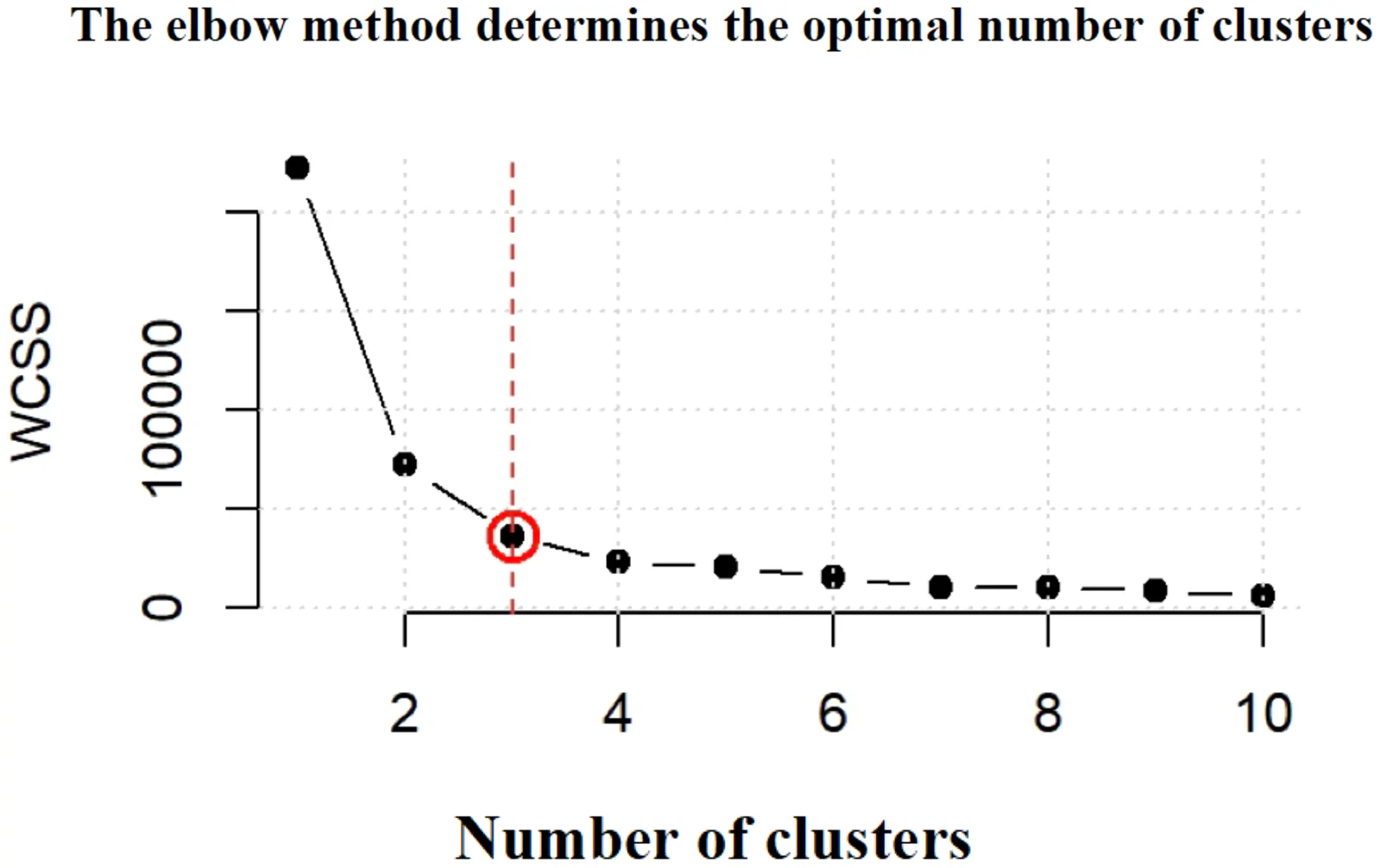
The elbow method determines the number of clusters when the body type is insufficient.

To describe subgroups within the body-dissatisfaction distribution, the number of clusters was selected using the elbow method. The within-cluster sum of squares showed an inflection at three clusters, and a three-cluster solution was adopted (**Figure 3**). K-means clustering was then applied to classify participants into three body-dissatisfaction subgroups. The elbow criterion is a heuristic; the three-cluster solution is a descriptive partition of a continuous variable and does not imply the existence of discrete latent classes.

The three body-dissatisfaction clusters (‘low’, ‘moderate’ and ‘high’) were cross-tabulated with the two BMI groups defined by the supplementary ROC-derived cut-off (‘low BMI’ and ‘high BMI’). A χ² test indicated that the distribution of body-dissatisfaction clusters differed between the two BMI groups (**Table 9**). This reflects the fact that measured BMI enters the calculation of the body-dissatisfaction score, so the two classifications are not independent; the association should therefore be read as descriptive rather than as an independent empirical finding.

**Table 9:** Cross-tabulation of different BMI categories and body dissatisfaction groups.

	Body dissatisfaction classification			Total	*χ²*	P
BMI classification	Low dissatisfaction	Medium dissatisfaction	High dissatisfaction			
Low BMI	0	4,116	1,030	5,146	904.283	<0.01
High BMI	453	1,766	563	2,782		
Total	453	5,882	1,593	7,928		

Cross-classifying the two BMI groups with the three body-dissatisfaction clusters yielded five non-empty strata (the low-BMI, low-dissatisfaction combination contained no participants). The observed prevalence of depressive symptoms and its Wilson 95% confidence interval within each stratum are reported in **Table 1**. One-way ANOVA indicated differences in mean SDS scores across strata. Because the BMI cut-off, the cluster boundaries and the outcome classification were all derived from this dataset, the values in **Table 1** are observed sample proportions and not validated risk estimates.

## 4 Discussion

This cross-sectional study examined the associations of BMI and body dissatisfaction with depressive symptoms among Chinese university students and found that both departed from linearity. In an exploratory decomposition, the indirect component acting through body dissatisfaction was larger at lower BMI values and attenuated as BMI increased. Cross-classifying BMI groups with body-dissatisfaction clusters produced strata with markedly different observed prevalences of depressive symptoms. Because body dissatisfaction was operationalised as a function of measured BMI and the design was cross-sectional, these results are interpreted below as statistical associations and as a description of the observed data, not as evidence for a psychological mechanism.

In this study, a preliminary analysis using a multiple linear regression model indicated that BMI was positively and linearly associated with depression (**Table 3**). However, the core logic of linear regression is to fit the ‘average effect’ between variables through a single slope (23). In our sample, 16.45% of participants were underweight (BMI < 18.5 kg/m^2^) and 28.51% were overweight or obese (BMI ≥ 24 kg/m^2^) (**Table 2**). A linear model cannot accurately capture the fundamentally different associations between BMI and depression in these two groups; it can only reflect overall trends. Therefore, we constructed an RCS model to model the potential relationship between BMI and depression more flexibly. The results indicated that the relationship between BMI and odds of clinically significant depressive symptoms was not simply linear, but rather exhibited a curve whose slope gradually flattened (**Figure 2**; **Table 5**).

Numerous epidemiological studies have reported a positive association between BMI and depression; a meta-analysis, for example, reported an association between high BMI and depression (24). Several earlier studies modelled this association as linear (25, 26). The present findings are consistent with a positive association and additionally indicate departure from linearity across the BMI range. This is in keeping with a large cross-sectional study of approximately one million adolescents aged 11 to 15 years, which reported a U-shaped association between BMI and mental health problems, with elevated risk in both underweight and obese groups (27). In the present sample, the estimated odds of depressive symptoms rose more steeply in the upper part of the BMI range. One possible interpretation is that body-image evaluation differs across sociocultural contexts, and it has been reported that body-image concerns are prevalent among Chinese university students (28). However, no measure of sociocultural pressure, appearance-related media exposure or internalised aesthetic standards was collected in this study, and this interpretation is therefore offered as a hypothesis for future testing rather than as an explanation supported by the present data.

As shown in **Figure 1** and **Table 4**, a non-linear association was observed between body dissatisfaction and the binary depressive-symptom outcome (SDS standard score >= 53). Below a body-dissatisfaction score of approximately 3.58, the estimated odds ratio rose steeply with increasing dissatisfaction. Across the middle of the range, the estimated odds ratio declined, and at higher values it rose again. This pattern refers to the binary outcome rather than to the continuous SDS score. The exploratory decomposition (**Table 8**) showed that the estimated indirect association through the discrepancy-based body-dissatisfaction score decreased as BMI increased, which is statistically consistent with the flattening of the BMI-depressive symptom curve. Because measured BMI is contained within the body-dissatisfaction indicator, part of this pattern may reflect mathematical dependence and shared variance rather than an independent psychological pathway; the findings are accordingly presented as an exploratory statistical decomposition. A longitudinal cohort study of British children has reported prospective pathways linking childhood BMI, body dissatisfaction and adolescent depression (29). At the lower end of the BMI range, a low BMI may itself be evaluated unfavourably, and in some settings has been reported to carry adverse social connotations (30), which could give rise to body dissatisfaction. The RCS curve for body dissatisfaction rises most steeply at its lower end, and the larger estimated indirect association at low BMI values is consistent with this feature of the curve. Whether this reflects self-criticism, social comparison or another process cannot be determined from the present data, and no such process was measured.

Across the middle of the body-dissatisfaction range, the estimated association with depressive symptoms does not increase monotonically along the RCS curve but shows a temporary decline. This should not be interpreted as evidence that increasing body dissatisfaction protects against depressive symptoms. It may reflect uneven data density across the range, uncertainty in the fitted estimates, or heterogeneity among participants. In addition, the absolute discrepancy score combines participants whose measured BMI exceeds their ideal BMI with those whose measured BMI falls below it; these two forms of discrepancy may correspond to different body-image concerns and need not have identical associations with depressive symptoms. The intermediate decline is therefore treated as a feature of the fitted curve in this sample, and no psychological interpretation is attached to it.

At higher BMI values, the estimated indirect association through body dissatisfaction was smallest. A 2025 study of overeating reported that, in the pathways examined, the direct contributions of dieting and emotional eating predominated, while the contribution of body dissatisfaction was comparatively weaker (31). Higher BMI may also be accompanied by physiological and social exposures that were not assessed here: obesity-related low-grade inflammation, physical or functional limitation, and external or internalised weight stigma have each been linked to depressive symptoms (32–34). Any of these could contribute to depressive symptoms alongside subjective body dissatisfaction and could therefore reduce its relative statistical contribution at higher BMI values. Because inflammatory markers, functional status, eating behaviour and weight stigma were not measured in this study, these remain untested hypotheses; the present data establish only that the estimated indirect component was smaller at higher BMI values.

The observed pattern is compatible with the view that individuals at different points of the BMI and body-dissatisfaction distributions may not be well served by a single approach. Any such implication is, however, indirect: the present study did not evaluate an intervention, did not follow participants over time, and cannot establish that altering body dissatisfaction would alter depressive symptoms. The findings are best regarded as a basis for designing longitudinal and intervention studies rather than as a guide to current practice.

**Figure 4:**
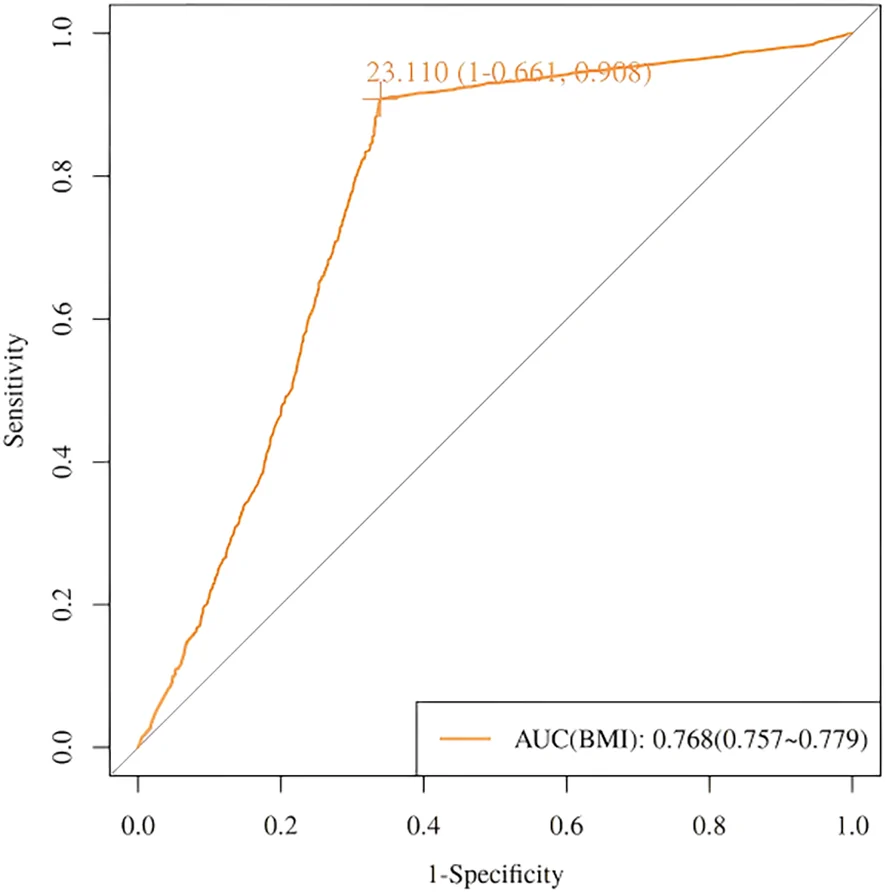
Receiver operating characteristic curve for the discrimination of clinically significant depressive symptoms by BMI.

As a separate supplementary analysis, ROC curve analysis was performed to evaluate the ability of BMI to distinguish participants with and without clinically significant depressive symptoms. The cutoff value that maximized the Youden index was 23.11 kg/m^2^ (**Figure 4**). Unlike the RCS reference value of 21.5 kg/m^2^, which was used only to normalize the odds ratio to 1, the ROC-derived cutoff was selected to optimize the combined sensitivity and specificity for the binary depressive-symptom outcome. Therefore, the two values have different statistical purposes and should not be interpreted as interchangeable thresholds.

The area under the ROC curve was 0.768. BMI is not a diagnostic or screening marker for depression, and this value should not be read as indicating that BMI is an accurate predictor of depressive symptoms; it describes only the extent to which BMI separated participants above and below the SDS screening cut-off in this particular sample. The cut-off of 23.11 kg/m² was derived from the same data used to evaluate it, was not tested in any independent sample, and is expected to differ in other populations, in samples with a different prevalence of depressive symptoms, and under a different outcome definition. When the ROC-derived cut-off was superimposed descriptively on the RCS curve, it fell in a region where the estimated odds of depressive symptoms were increasing rather than in the later plateau region (**Figure 2**); this comparison is descriptive only, since the cut-off was selected for classification performance whereas the spline describes the estimated dose-response association relative to the prespecified reference value. The cut-off should not be interpreted as a biological threshold, as an inflection point of the RCS curve, or as a BMI value at which depressive-symptom risk begins to increase, and it is not proposed for use in screening.

Cross-classifying BMI groups with body-dissatisfaction clusters produced five strata whose observed prevalence of depressive symptoms differed substantially (**Table 1**). The cross-tabulation in **Table 9** shows that body-dissatisfaction clusters were unevenly distributed across BMI groups, which is consistent with the social comparison framework linking weight-related characteristics to body-image perception (35) but is also an arithmetic consequence of deriving the dissatisfaction score from measured BMI. In the present sample, all participants in the high-BMI, moderate-body-dissatisfaction stratum met the SDS screening cut-off. This is an observed sample proportion, not a probability and not a validated individual-level prediction; a proportion of 100% in a single stratum warrants particular caution, since it may reflect the small effective variation within that stratum, the derivation of stratum boundaries and outcome classification from the same dataset, or a combination of both. The present data do not permit the observed pattern to be attributed to emotional suppression, experiential avoidance, body-image coping, weight stigma or any other unmeasured factor, and no such attribution is made.

If externally validated, the matrix could potentially serve as an auxiliary component of a stepped digital triage process in university settings. Students could complete validated measures of depressive symptoms and body-image concerns through a secure campus platform, while the matrix could be used to identify students who may benefit from further assessment. It should not be used to generate a psychiatric diagnosis or to replace standardized screening and professional evaluation. Students with elevated screening results should be reviewed by trained campus counsellors or mental-health professionals, with clear referral procedures for severe symptoms or self-harm risk. Any future digital implementation would require independent validation, informed consent, privacy protection, transparent decision rules, and human oversight (36).

The low-BMI, high-body-dissatisfaction stratum showed a high observed prevalence of depressive symptoms (61.3%, 95% CI 58.2 to 64.2) despite the absence of objective weight excess. Body-image concern in the absence of overweight has been discussed in terms of internalised aesthetic standards and negative self-evaluation (37) and in terms of the tripartite influence model, in which appearance-related pressures from parents, peers and the media are internalised and sustain appearance monitoring and unfavourable social comparison (38). These frameworks were not tested here, as no measure of internalisation or social comparison was collected. The descriptive point supported by the present data is narrower but still relevant: elevated depressive-symptom prevalence was not confined to participants with high BMI, and low BMI combined with high body dissatisfaction was accompanied by a comparable prevalence.

The high-BMI, high-body-dissatisfaction stratum showed an observed prevalence of 58.8% (95% CI 54.6 to 62.9), which overlapped with that of the low-BMI, high-body-dissatisfaction stratum (61.3%, 95% CI 58.2 to 64.2). High BMI was therefore not accompanied by a higher observed prevalence than low BMI once body dissatisfaction was high. An intervention study among overweight young adults reported that improvement in problem-solving skills predicted reduction in depressive symptoms (39), which raises the possibility that coping and adaptation differ across these strata; the present cross-sectional data contain no measure of coping and cannot test this possibility. The comparison of the two strata is descriptive, and the overlapping confidence intervals should be borne in mind.

The low-BMI, moderate-body-dissatisfaction stratum was the largest in the sample and had the lowest observed prevalence of depressive symptoms (14.4%, 95% CI 13.4 to 15.5). That moderate body dissatisfaction was the modal category in this sample indicates that some degree of body-image concern was common among the students surveyed. Whether body-image education would alter depressive-symptom prevalence in this group is not addressed by the present design and would require intervention research.

In summary, this cross-sectional study describes non-linear associations of BMI and body dissatisfaction with depressive symptoms in a large sample of Chinese university students, together with an exploratory decomposition indicating that the indirect component through body dissatisfaction was larger at lower BMI values. The accompanying stratification matrix is a description of the observed data and is not a validated screening tool. Its value lies in identifying combinations of BMI and body dissatisfaction that merit prospective investigation, and any application to campus mental-health screening would require independent validation.

## 5 Limitations

This study has several limitations. First, the design is cross-sectional, so temporal ordering among BMI, body dissatisfaction and depressive symptoms cannot be established. Body dissatisfaction may follow from BMI, may influence eating behaviour and thereby BMI, and may be affected by depressive symptoms themselves. The decomposition reported here is therefore an exploratory statistical partition of association and not a test of mediation, which requires temporal ordering that the present design cannot provide. Longitudinal designs with repeated measurement of all three constructs are needed.

Second, and most importantly, the mediator is mathematically derived from the exposure. Body dissatisfaction was operationalised as the absolute difference between measured BMI and self-reported ideal BMI, so measured BMI is embedded within the mediator and the two are not statistically independent. Part of the estimated indirect association may therefore arise from this structural dependence rather than from any psychological pathway. In addition, the absolute difference captures the magnitude but not the direction of discrepancy, so participants who wish to weigh less are combined with those who wish to weigh more, although these groups may differ in their body-image concerns and in their association with depressive symptoms. Future work should use validated multidimensional body-image instruments that are measured independently of BMI.

Third, residual confounding is likely to be substantial. The regression models were adjusted for age and sex only. Depressive symptoms are associated with many factors that were not measured in this study, including socioeconomic status, sleep quality, physical activity, dietary habits, smoking, alcohol consumption, anxiety, chronic disease, perceived stress, academic pressure, social support, social media use and appearance-related sociocultural pressure. Several of these are also associated with BMI and with body dissatisfaction and could therefore account for part of the associations reported here. The adjusted estimates should not be regarded as approximating causal effects, and the magnitude and direction of residual confounding cannot be quantified from the present data.

Fourth, the five-stratum matrix was developed and evaluated in the same dataset. No internal validation, cross-validation, bootstrap optimism correction or external validation was performed, and no independent sample was available. The stratum-specific prevalences and the apparent separation between strata are therefore optimistic, and the matrix is presented as an exploratory description rather than as a prediction model. The observation of a 100% prevalence in one stratum further indicates that stratum-specific estimates should be treated with caution, as such a value cannot be expected to reproduce in an independent sample. Independent validation is a prerequisite for any screening or triage application.

Fifth, the ROC analysis is supplementary and of limited clinical relevance. BMI is not intended as a diagnostic or screening marker for depression, and the cut-off of 23.11 kg/m² is a sample-specific value obtained by maximising the Youden index in these data. It has not been validated, would be expected to shift with the prevalence of depressive symptoms and with the outcome definition, and should not be used as a screening threshold.

Sixth, features of the fitted spline curves should be interpreted with caution. Regions in which the estimated odds ratio changes direction may correspond to parts of the distribution with sparse observations, and may reflect the flexibility of the spline basis and the width of the confidence bands rather than a substantive change in the underlying association. Psychological interpretations of individual curve features are not supported by the present design. Relatedly, 5.0% of BMI observations were winsorised, which compresses the tails of the distribution and may attenuate the estimated associations at the extremes of the BMI range; no analysis was repeated on untreated data, so the sensitivity of the results to this decision has not been quantified.

Seventh, the findings may not generalise beyond the setting studied. Participants were full-time university students at eight institutions in five Chinese provinces and municipalities, and are not representative of Chinese young adults generally, still less of populations elsewhere. Ideal body size, the meaning attached to thinness and to fullness, and the acceptability of reporting psychological distress vary across cultural settings. Because body dissatisfaction here was defined relative to a self-reported ideal BMI, its distribution and its association with depressive symptoms would be expected to differ where prevailing body ideals differ. The associations reported here should therefore be re-examined before being applied outside comparable Chinese university populations.

Finally, depressive symptoms were assessed with the Zung Self-Rating Depression Scale, a self-report screening instrument rather than a diagnostic interview. The binary outcome defined by an SDS standard score of 53 or above therefore denotes symptoms at or above a screening threshold and not a diagnosis of depressive disorder; prevalence figures reported here are prevalences of screening positivity. The results may also be affected by social-desirability bias, individual differences in item interpretation and culturally patterned response styles. Future studies should combine validated self-report instruments with structured clinical interviews.

## 6 Conclusions

In this cross-sectional study of Chinese university students, BMI and body dissatisfaction were both non-linearly associated with depressive symptoms. An exploratory decomposition indicated that the indirect component of the BMI-depressive symptom association acting through body dissatisfaction was larger at lower BMI values and attenuated as BMI increased. Because the body-dissatisfaction indicator is mathematically derived from measured BMI and the design is cross-sectional, these results describe statistical associations and do not establish a mediating mechanism. The supplementary ROC analysis and the five-stratum descriptive matrix were derived and evaluated in the same dataset without validation and are reported as exploratory; neither is proposed for screening use. The main contribution of this study is to show that the association between BMI and depressive symptoms in this population is not adequately represented by a linear model, and that both low and high BMI accompanied by body dissatisfaction warrant attention in future longitudinal research.

## Data Availability

The raw data supporting the conclusions of this article will be made available by the authors, without undue reservation.
